# Chronic active Epstein–Barr virus-associated secondary hemophagocytic lymphohistiocytosis in pregnancy: a case report

**DOI:** 10.1186/s12884-021-04150-4

**Published:** 2021-10-07

**Authors:** Masaya Takahashi, Shintaro Makino, Hiroko Iizuka, Masaaki Noguchi, Koyo Yoshida

**Affiliations:** 1 Department of Obstetrics and Gynecology, Juntendo University Urayasu Hospital, Chiba Urayasu City, Japan; 2 Department of Hematology, Juntendo University Urayasu Hospital, Urayasu City, Chiba Japan

**Keywords:** Secondary hemophagocytic lymphohistiocytosis, Hemophagocytosis, Epstein–Barr virus, Pregnancy, Case report

## Abstract

**Background:**

Secondary hemophagocytic lymphohistiocytosis (sHLH) is a rare and fatal disease characterized by uncontrolled immune cell activation that can lead to a cytokine storm. Unfortunately, this condition can occur even during pregnancy, threatening both maternal and fetal lives.

**Case presentation:**

A 23-year-old nulliparous woman at 26 weeks of gestation presented with continuous fever, coughing, and sore throat. Upon arrival at our hospital, her temperature was >38°C and laboratory findings indicated cytopenia (neutrophil count, 779/μL; hemoglobin level, 10.2 g/dL; platelet count, 29,000/μL), elevated ferritin level (1,308 ng/mL), and elevated soluble interleukin-2 receptor level (11,200 U/mL). Computed tomography showed marked splenomegaly. Bone marrow examination revealed hemophagocytosis, and blood examination showed a plasma Epstein–Barr virus (EBV) DNA level of 8.9 × 10^5^ copies/μg. The monoclonal proliferation of EBV-infected T cells was confirmed by Southern blotting, and the patient was diagnosed with chronic active EBV-associated sHLH and T-cell lymphoproliferative disease. Immediately after admission, the patient’s condition suddenly deteriorated. She developed shock and disseminated intravascular coagulation, requiring endotracheal intubation along with methylprednisolone pulse and etoposide therapy. Although the patient recovered, she delivered a stillborn baby. After delivery, she was treated with reduced-dose dexamethasone, etoposide, ifosfamide, and carboplatin (DeVIC) and steroid (dexamethasone), methotrexate, ifosfamide, L-asparaginase, and etoposide (SMILE) chemotherapies. Five months after diagnosis, she received human leukocyte antigen-haploidentical allogeneic bone marrow transplantation from her sister. She remains in remission for 5 months from the time of transplantation to the present.

**Conclusions:**

sHLH, which may cause maternal and fetal death, should be carefully considered in critically ill pregnant women, particularly those presenting with continuous fever and cytopenia.

## Background

Hemophagocytic lymphohistiocytosis (HLH) is an extremely rare and life-threatening disease characterized by excessive immune cell activation that may cause a cytokine storm [1–3], leading to severe tissue damage, cell death, and multiple organ failure [4]. HLH can be classified as either primary or secondary. Primary HLH is mainly observed as familial HLH, which develops in childhood in association with gene mutations, and can also present as inherited immune deficiency syndromes [5]. Secondary HLH (sHLH), also known as acquired HLH, is mainly caused by infections (50.4%), malignancies (47.7%), and autoimmune diseases (12.6%) [1, 6]. The epidemiology of HLH varies considerably depending on the population heterogeneity and variable underlying triggers [6]. In fact, sHLH is one of the most fatal diseases in adults, with a mortality rate of up to 41%, but the prognosis also depends on the underlying triggers [6]. Owing to potential malignancy, bone marrow examination (BME) should be performed to detect underlying tumors [1, 7]. Unfortunately, the fatal condition caused by sHLH can occur even during pregnancy and threaten both maternal and fetal lives via cytopenia-related complications, multiple organ failure, and aberrant cytokine storms [4, 8–10]. Considering its extremely rare occurrence during pregnancy, the exact etiology and appropriate management of sHLH during pregnancy remain unclear [8–11].

As pregnancy induces changes in clinical features and laboratory findings that appear similar to the clinical findings of sHLH, differentiating sHLH from other conditions becomes difficult [11–14]. Further, considering the likelihood of death because of delayed diagnosis and treatment, early diagnosis with immediate therapeutic intervention, including steroids, chemotherapies (such as etoposide), or hematopoietic stem cell transplantation (HSCT), is vital for treating sHLH [1, 11, 15]. The current report focuses on a novel case diagnosed with chronic active Epstein–Barr virus (CAEBV)-associated sHLH and T-cell lymphoproliferative disease during pregnancy.

## Case presentation

**Fig. 1:**
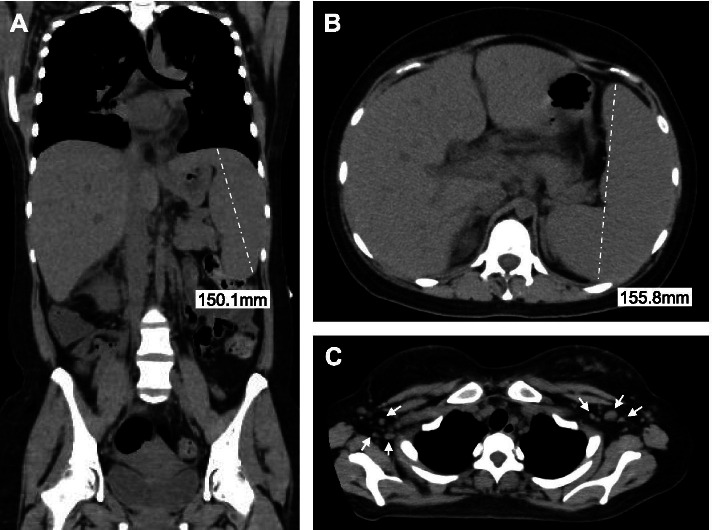
Computed tomography from the thorax to the pelvis showing remarkable splenomegaly and bilateral multiple axillary lymphadenopathy. **A** and **B** show splenomegaly. **C** shows bilateral multiple axillary lymphadenopathy (arrows)

**Fig. 2:**
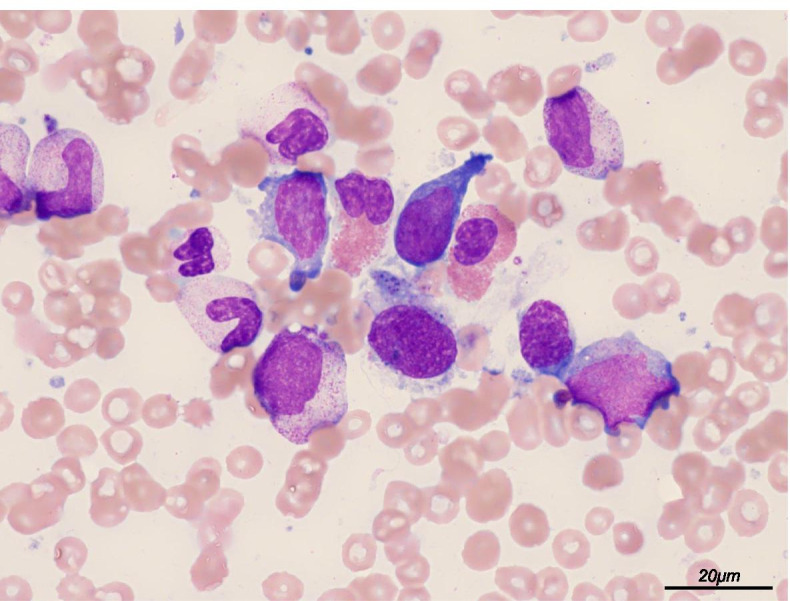
Hematoxylin and eosin staining of bone marrow aspirate showing hemophagocytosis. Scale bar, 20 μm

**Fig. 3:**
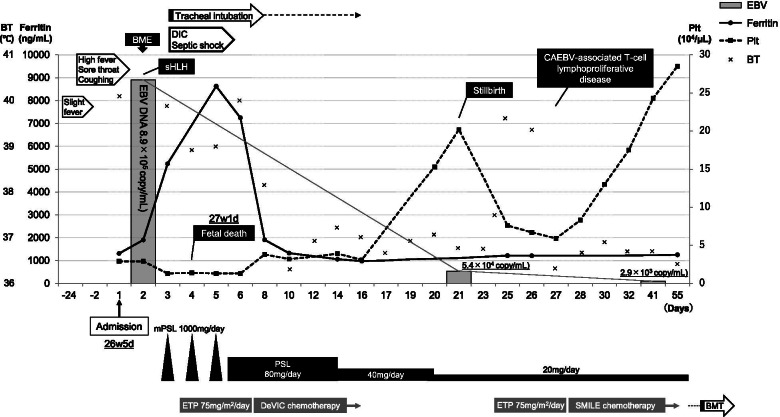
Clinical course of the patient. The detailed clinical course, therapeutic interventions, and laboratory findings (EBV DNA level, ferritin level, and platelet count) are shown. Abbreviations: BT, body temperature; BME, bone marrow examination; EBV, Epstein–Barr virus; sHLH, secondary hemophagocytic lymphohistiocytosis; DIC, disseminated intravascular coagulation; CAEBV, chronic active EBV; Plt, platelet; PSL, prednisolone; mPSL, methyl PSL; ETP, etoposide; DeVIC, dexamethasone, etoposide, ifosfamide, and carboplatin; SMILE, steroid (dexamethasone), methotrexate, ifosfamide, L-asparaginase, and etoposide; BMT, bone marrow transplantation

A 23-year-old pregnant nulliparous woman at 26 weeks of gestation without any past medical history visited a general physician complaining of continuous fever for 3 weeks with a 2-day history of recent high fever, coughing, and sore throat. No history of recent foreign travel was noted. After excluding coronavirus disease 2019, she was transferred to our hospital owing to her critical status and admitted to maternal fetal intensive care unit. Physical examination revealed a fever (40.2°C) and tachycardia (142 beats/min) but normal blood pressure (102/56 mmHg) and respiratory rate (19/min). An initial nonstress test of the fetus showed normal baseline heart rate (140 beats/min) with moderate variability and acceleration in the absence of any deceleration. The fetal biophysical profile scoring indicated full marks. Laboratory tests showed cytopenia (neutrophil count, 779/μL; hemoglobin level, 10.2 g/dL; and platelet count, 29,000/μL), elevated C-reactive protein level (16.1 mg/dL), elevated ferritin level (1,308 ng/mL), and elevated soluble interleukin (IL)-2 receptor (sIL-2R) level (11,200 U/mL) (Table 1). No liver dysfunction was observed. Computed tomography from the thorax to the pelvis showed remarkable splenomegaly and bilateral multiple axillary lymphadenopathies (Fig. 1). Taken together, some underlying hematological etiology was suspected. Therefore, a hematologist was consulted soon after admission. BME on day 2 of hospitalization revealed hemophagocytosis (Fig. 2). Considering fever, splenomegaly, bicytopenia, hyperferritinemia, elevated sIL-2R level, and hemophagocytosis, the patient satisfied the clinical diagnostic criteria of sHLH (six out of eight). Thus, the patient was immediately started on methylprednisolone pulse (1,000 mg/day) and etoposide therapy (75 mg/m^2^/day) for hemophagocytosis. Broad spectrum antibiotics were also administered intravenously for treating sepsis of unknown origin. Owing to severe cytopenia and coagulation abnormality, the patient required a large amount of supporting blood products. According to the hematologist, changes in body temperature, platelet count, and serum ferritin levels indicated sHLH (Fig. 3). On day 3 of hospitalization, her serum ferritin level increased to >5,000 ng/mL. At this point, her condition suddenly deteriorated, and she developed shock and disseminated intravascular coagulation (DIC), prompting her transfer to the intensive care unit for endotracheal intubation and mechanical ventilation. Owing to high fetal mortality rates among the few studies on the prognosis of pregnancy-associated sHLH, an emergency cesarean section was considered. However, after discussions with the hematologist and emergency physician, we decided against the procedure because of the extreme critical status of the patient characterized by severe shock and DIC, preventing surgery. Our discussions also prompted us to prioritize maternal life considering her poor condition and the high mortality rate of sHLH. Throughout the disease course, obstetricians could only perform frequent assessments of fetal well-being via transabdominal ultrasound. Unfortunately, spontaneous fetal demise occurred at 27 weeks of gestation (day 4 of hospitalization).

**Table 1:** Laboratory test on admission day

Laboratory value	Value	Normal range or control	Unit
WBC	19	40–80	10^2^/μL
Lymphocytes	51	25–45	%
Neutrophils	41	37–72	%
Neutrophil count	779	-	/μL
Hb	10.2	12–16	g/dL
Plt	2.9	15–35	10^4^/μL
CRP	16.1	0–0.3	mg/dL
BUN	9	8–22	mg/dL
Creatinine	0.59	0.47–0.79	mg/dL
Total bilirubin	0.6	0.2–1.3	mg/dL
AST	42	13–33	IU/L
ALT	11	8–42	IU/L
LDH	630	119–229	IU/L
Ferritin	1,308	30–400	ng/mL
Triglyceride	234	30–149	mg/dL
sIL-2R	11,200	157–474	U/mL
PT/INR	0.97	-	INR
APTT	37.5	30.2	s
Fibrinogen	453	150–400	mg/dL
EBV DNA	8.9 × 10^5^	-	copies/mL

*Abbreviations*: *WBC* white blood cell, *Hb* hemoglobin, *Plt* platelet, *CRP* C-reactive protein, *BUN* blood urea nitrogen, *AST* aspartate transaminase, *ALT* alanine transaminase, *LDH* lactate dehydrogenase, *sIL-2R* soluble interleukin-2 receptor, *PT-INR* prothrombin time/international normalized ratio, *APTT* activated partial thromboplastin time, *EBV* Epstein–Barr virus

**Fig 4:**
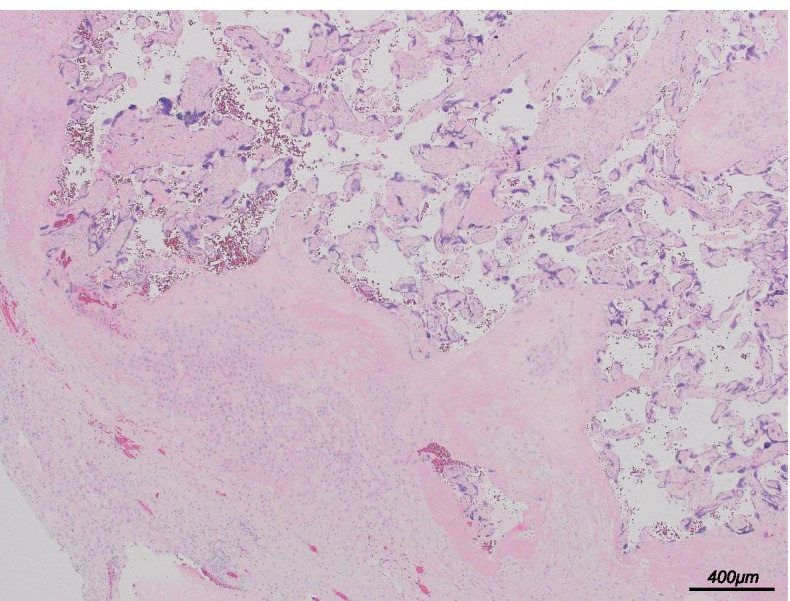
Hematoxylin and eosin (HE) staining of the placenta. HE staining. Scale bar, 400 μm

A critically high level of plasma EBV DNA level was observed on day 2 of hospitalization (8.9 × 10^5^ copies/μg), suggesting EBV infection. Her peripheral blood examination showed positive immunoglobulin (Ig)G antibodies to the EBV early antigen and capsid antigen as well as negative IgM antibodies to the EBV capsid antigen, suggesting EBV infection reactivation. Southern blotting demonstrated monoclonal proliferation of EBV-infected T cells, whereas immunophenotyping was strongly positive for CD_3_, CD_8_, cytotoxic T cell-derived granzyme B, and EBV-encoded small RNA, suggesting the neoplastic proliferation of EBV-infected T cells. Based on these findings, the patient was diagnosed with CAEBV-associated T-cell lymphoproliferative disease. Patients with CAEBV often develop EBV-positive T or natural killer cell lymphomas. CAEBV is refractory to conventional chemotherapy and has a poor prognosis. At present, HSCT is the only curative therapy for CAEBV. Although steroid (dexamethasone), methotrexate, ifosfamide, L-asparaginase, and etoposide (SMILE) chemotherapy is the standard therapy for this condition, this could not be administered because of her poor health status. Thus, she was treated with reduced-dose dexamethasone, etoposide, ifosfamide, and carboplatin (DeVIC) chemotherapy along with prednisolone (PSL) therapy (days 6–13, PSL 80 mg/day; day 14–19, PSL 40 mg/day; after day 20, PSL 20 mg/day). Fortunately, she responded positively to these treatments. Her serum ferritin levels rapidly decreased to approximately 1,000 ng/mL soon after initiating methylprednisolone pulse and etoposide therapy. She became afebrile and no longer needed endotracheal intubation. Her platelet count gradually increased to normal levels, and her clinical condition improved on day 21 of hospitalization after reduced-dose DeVIC chemotherapy. On day 21 of hospitalization, plasma EBV DNA level decreased to 5.4 × 10^4^ copies/μg, whereas other clinical findings suggested recovery from DIC and chemotherapy-derived bone marrow suppression. Thus, labor induction was planned as continuous pregnancy with a stillborn baby promotes DIC. Of note, the mere insertion of a stick for cervical dilation induced spontaneous labor, and she delivered an 840-g female baby who did not have any external malformations. As the patient and her family refused an autopsy of the baby, no further details could be determined. Placental and umbilical cord pathology revealed no abnormalities, such as chorioamnionitis (Fig. 4). Moreover, EBV-encoded small RNA in situ hybridization showed no EBV-infected lymphocytes in the placenta. Considering that no remarkable findings related to fetal death were noted and that the clinical features of sHLH were associated with hypercytokinemia, which leads to fetal death, we suspected that fetal demise was caused by excessive cytokine activity. To confirm this, maternal serum interferon-γ (IFN-γ) levels at admission were measured, showing marked elevation up to 380 IU/mL.

On day 25 of hospitalization, she suddenly became febrile again and her condition deteriorated with progressive cytopenia, suggesting that reduced DeVIC chemotherapy was ineffective. Thus, we started second etoposide therapy (75 mg/m^2^/day) and SMILE chemotherapy. She responded to the therapies, and her platelet count returned to normal with a robust decline in plasma EBV DNA levels. For further treatment, she was transferred to another hospital that specializes in treating CAEBV. Five months after diagnosis, she received human leukocyte antigen-haploidentical allogeneic bone marrow transplantation from her sister. She remains in remission for 5 months from the time of transplantation to the present.

## Discussion and conclusions

The current case report involved a novel and critical malignant case of CAEBV-associated sHLH during pregnancy with rapid deterioration, shock, and DIC. Considering the high maternal and fetal mortality rates among such cases, careful discussion regarding maternal and fetal life is needed when obtaining informed consent. In the present case, priority was given to maternal treatment because of her critical clinical status. In pregnant women diagnosed with sHLH, obstetricians are recommended to pay special attention to the fetus via frequent transabdominal ultrasounds and/or nonstress tests to assess fetal heartbeat and growth to appropriately discuss the possible next steps for the mother and baby.

This case highlights the importance of including sHLH as a differential diagnosis in critically ill pregnant women as this condition, if not detected, could be fatal for both the mother and fetus. While early diagnosis with immediate therapeutic intervention in the current case led to recovery, the patient still delivered a stillborn baby.

The pathophysiology of sHLH involves uncontrolled inflammatory responses with the excessive activation of immune cells, including IFN-γ-producing T cells [4]. Highly elevated IFN-γ promotes phagocytosis, leading to the overproduction of proinflammatory cytokines [4]. In the current case, the patient’s serum IFN-γ levels upon admission were markedly elevated, which may have caused severe tissue damage and resulted in multiple organ failure. During normal pregnancy, CD_4_^+^ helper T (Th) cells typically shift from Th1 to Th2 dominance for immune tolerance to the genetically foreign fetus; thus, humoral immunity is dominant over cell-mediated immunity [16]. However, in patients with sHLH, this homeostasis of immune cells during pregnancy collapses, leading to aberrant immune cell activities. In general, EBV, which was observed in the present case, mainly infects CD_8_^+^ T cells (also known as killer T cells) [17]. EBV-infected cytotoxic CD_8_^+^ T cells can impair their proper function, whereas activated CD_8_^+^ T cells suppress humoral immunity and drive continuous T cell hyperactivation, which is evident in sHLH [17].

Clinical findings are important to assess the pathology of sHLH. According to previous reports, peak serum ferritin levels >10,000 ng/mL are 90% sensitive and 96% specific for diagnosing sHLH [18]. Meanwhile, other studies have shown that serum sIL-2R levels (>2,515 U/mL) are 100% sensitive and 72.5% specific [19]. In our patient, the peak levels of serum ferritin and serum sIL-2R were 8,630 ng/mL (on day 5 of hospitalization) and 11,200 U/mL (on day 1 of hospitalization), respectively. Delayed sHLH diagnosis can result in death; however, we consulted the hematologist regarding her bicytopenia, which prompted timely diagnosis and proper therapeutic interventions (including steroids, etoposide, and other chemotherapies).

Only 44 cases of pregnancy-associated sHLH have been previously documented [8–10, 15, 20–24]. In these reports, maternal and fetal mortality were strikingly high at 20.5% (9 cases) and 27.3% (12 cases), respectively. Moreover, three cases of maternal death were caused by EBV infection. By contrast, an ex vivo study in humans by Aaltonen et al. demonstrated no evidence of transplacental transfer of several cytokines [25]. Further, some chronic inflammatory conditions, including human immunodeficiency virus 1 or hepatitis B virus infection, lead to higher cytokine levels in the umbilical cord blood that can affect fetal immune responses. In such cases, the fetus can be influenced by maternal cytokines through unknown mechanisms, possibly via the placenta [26, 27]. As our case received prompt and precise respiratory and circulatory support, we speculated that a maternal hyper-cytokine storm affected the fetus and caused fetal demise. Therefore, suppressing excessive immune response as soon as possible is crucial for preventing both maternal and fetal deaths.

Although the exact etiology of pregnancy-induced sHLH remains unclear, a transition of fetal-derived components, including trophoblast debris and soluble RNA or DNA of fetal origins, into the maternal blood circulation might lead to abnormal immunological responses and trigger systemic inflammation, thereby causing cytokine storms [28, 29]. Previous reports showed that sHLH remission occurs after pregnancy termination via abortion or delivery [8, 28]; however, it remains unclear whether the termination itself contributes to sHLH remission. Indeed, other reports showed that deceased patients with sHLH showed progressive deterioration even after pregnancy termination [30], suggesting the difficulty of controlling cytokine storms once activated; this leads to a further hyperimmune state. Thus, preventing uncontrolled aberrant cytokine storms with rapid therapeutic interventions, such as steroids or etoposide, is imperative.

BME is essential in patients suspected of sHLH, even during pregnancy, to detect hemophagocytosis and/or underlying malignancy. A recent study reported that BME did not reveal hemophagocytosis in approximately 30% of patients with sHLH and that hemophagocytosis occasionally could not be detected in the early stage of sHLH [7]. Thus, apart from repeated BME, determining whether clinical features and laboratory findings fulfill the sHLH criteria [31] and HScore, a tool used for diagnosing sHLH [32], is also required. Moreover, excluding the diagnosis of malignancy is crucial as patients with sHLH and lymphoma show poor prognosis. The overall survival rate of lymphoma-related sHLH is only 8% [33]. Most importantly, the treatment plan for sHLH differs depending on whether malignancy exists. By establishing the diagnosis of malignancy, chemotherapy or bone marrow transplantation can be initiated.

Regarding lymphoma, it is important to be conscious of its transmission. Two cases of fetal transmission of maternally-derived lymphoma have been previously reported, with the infants presenting with symptoms similar to sHLH, including fever, tachycardia, hepatomegaly, and splenomegaly, within the first year of life [34, 35]. As the direct invasion of activated T cells to the placenta was not observed in the present case, we believe that no transmission to the fetus occurred. Hence, the close monitoring of newborn babies born to mothers with hematological malignancies is vital, particularly within the first year of life.

The etiology of lymphoma-related sHLH is another factor that should be considered. In the current case, sHLH symptoms appeared as a consequence of EBV reactivation that occurred during pregnancy. Haeri et al. who investigated the prevalence of EBV seropositivity in 64 healthy pregnant women [36], demonstrated that 22 (35%) women showed EBV reactivation during pregnancy, regardless of maternal age, race, parity, or insurance type [36]. Moreover, previous reports have clarified that EBV reactivation during pregnancy lead to poor obstetrical outcomes, such as fetal growth restriction [37], shorter pregnancy duration [38], and lower birth weight [38], despite not showing any symptoms. Thus, we recommend transabdominal ultrasound and/or nonstress tests to determine the presence of these complications.

As mentioned above, sHLH can occur even during pregnancy; thus, obstetricians could contribute to the early diagnosis of sHLH, provided that they have knowledge regarding its clinical features. Iron-deficiency anemia or gestational thrombocytopenia may progress with gestational age, the laboratory findings of which sometimes present simultaneously with bicytopenia, thereby satisfying the diagnostic criteria of sHLH [31]. Moreover, a progressive increase in serum triglyceride levels, one of the criteria of sHLH, has been observed during pregnancy. However, it might be confusing to discriminate between normal pregnancy status and sHLH only by these laboratory findings. Although some similarities exist between sHLH and pregnancy-related findings, the typical laboratory findings of sHLH, including neutropenia, low fibrinogen levels, and hyperferritinemia, are uncommon during pregnancy. Thus, in critically ill pregnant women with unremitting fever and bi- or pancytopenia (particularly neutropenia), sHLH should be considered. It is necessary to rapidly assess for splenomegaly and ferritin, fibrinogen, and soluble IL-2 receptor levels as well as determine whether the clinical features and laboratory findings satisfy the sHLH criteria [31] and HScore [32].

Hemolysis, elevated liver enzymes, and low platelets (HELLP) syndrome should be carefully differentiated from sHLH. HELLP syndrome shares typical laboratory triads with sHLH; thus, differentiation between the two diseases can be confusing. In previous reports, four cases were misdiagnosed with HELLP syndrome instead of sHLH, and emergency cesarean section was performed. However, as their clinical condition did not improve even after delivery, the patients were rediagnosed with sHLH later [24, 33, 39, 40]. Therefore, it is important to consider sHLH when the patient shows no improvement even after pregnancy termination.

Suppressing uncontrolled cytokine storm along with the intervention for underlying diseases is essential for treating sHLH [1]. Steroids, intravenous Ig, cyclosporine A, and etoposide are often used to control the hyperinflammatory state [1]. However, no established treatment guidelines are available for sHLH during pregnancy while the appropriate treatment is still being highly debated. Physicians tend to hesitate while prescribing immunosuppressants or chemotherapy considering the potential harm to the fetus. In previous reports, 16 cases were treated with etoposide for sHLH during the perinatal period, among which 7 cases (including our case) were safely treated with etoposide during pregnancy with severe sHLH [10, 15, 21, 30, 41]. As sHLH during pregnancy often occurs in the second trimester [8–10, 15, 20–24], we primarily considered fetal toxicity rather than fetal teratogenicity. Moreover, given that sHLH during pregnancy has a high mortality rate, the maternal clinical condition can be prioritized rather than harm to the fetus [10, 15, 42].

With regard to the timing of delivery, bone marrow suppression in the mother and fetus should be considered. In recent years, salvage therapies using JAK1/2 inhibitor ruxolitinib or IL-1 receptor antagonist anakinra for sHLH during pregnancy have been found to be effective [21, 22]. However, given that only a few such cases were included, the beneficial or adverse effects of these drug treatments need further investigations.

This study has a limitation that should be addressed. In particular, the follow-up period was short. The 30-day mortality of primary and secondary HLH treated with various regimens is 20%–44%, whereas the overall mortality rate is 50%–75% [4]. Hence, the patient must be continuously observed for a long time.

In conclusion, obstetricians and hematologists should consider sHLH as a differential diagnosis in critically ill pregnant women.

## Data Availability

Anonymized data are available from the corresponding author upon reasonable request.

## Abbreviations

HLH: Hemophagocytic lymphohistiocytosis
sHLH: Secondary hemophagocytic lymphohistiocytosis
BME: Bone marrow examination
HSCT: Hematopoietic stem cell transplantation
CAEBV: Chronic active Epstein–Barr virus
IL: Interleukin
sIL-2R: Soluble IL-2 receptor
DIC: Disseminated intravascular coagulation
EBV: Epstein–Barr virus
Ig: Immunoglobulin
SMILE: Steroid (dexamethasone), methotrexate, ifosfamide, L-asparaginase, and etoposide
DeVIC: Dexamethasone, etoposide, ifosfamide, and carboplatin
PSL: prednisolone
IFN-γ: Interferon-γ
Th: Helper T cell
HELLP: Hemolysis, elevated liver enzymes, and low platelets
